# Three and two-dimensional cardiac mechanics by speckle tracking are predictors of outcomes in chagas heart disease

**DOI:** 10.1038/s41598-022-16379-w

**Published:** 2022-07-18

**Authors:** Viviane Tiemi Hotta, Maria Cristina Donadio Abduch, Marcelo Luiz Campos Vieira, Andrea de Andrade Vilela, Edimar Alcides Bocchi

**Affiliations:** 1 Clinical Unity of Cardiomyopathies, Instituto do Coração (InCor) do Hospital das Clínicas da Faculdade de Medicina da Universidade de São Paulo, Av. Dr. Enéas Carvalho de Aguiar, 44, Cerqueira Cezar, São Paulo, CEP: 05403/000 Brazil; 2 Echocardiography Sector, Fleury Medicina e Saúde, São Paulo, Brazil; 3 Echocardiography Sector, Instituto Dante Pazzanese de Cardiologia, São Paulo, Brazil; 4 Heart FailureDepartment, Instituto do Coração (InCor) do Hospital das Clínicas da Faculdade de Medicina da Universidade de São Paulo, São Paulo, Brazil

**Keywords:** Cardiology, Diseases, Medical research

## Abstract

Chagas disease (CD) is a neglected infectious disease associated with early mortality and substantial disability. Three-dimensional speckle tracking (3D STE) may play a role in the evaluation of CD. We aim to characterize new echocardiographic variables in patients with CD and to assess the hypothesis that 3D STE may predict outcomes. Seventy-two patients with CD were included. Clinical and conventional 2D and 3D STE analysis were performed. Patients were followed up for 60 months. Clinical events were defined as hospitalization for heart failure, complex ventricular arrhythmias, heart transplant and all-cause death. Seventy-two patients were recruited and enrolled in three groups: left ventricular ejection fraction (LVEF) < 0.40 (N = 22; reduced LVEF or rLVEF); 0.40 ≤ LVEF ≤ 0.50 (N = 10; mildly reduced LVEF or mrLVEF) and LVEF > 0.50 (N = 30; preserved LVEF or pLVEF). After a Cox model analysis, the top predictors of composite endpoints were 2D LV global longitudinal strain (GLS) ≤ − 11.3% (AUC = 0.87), 2D LV global circumferential strain (GCS) ≤  − 10.1% (AUC = 0.79), 3D LV GLS ≤ − 13% (AUC = 0.82), 3D LV area strain ≤ − 16% (AUC = 0.81) and right ventricle (RV) GLS ≤ − 17.2% (AUC = 0.78). Patients with CD and mrLVEF were morphologically similar to the rLVEF patients despite the benign evolution as the pLVEF group. RV GLS, 2D LV GLS, 2D LV GCS, 3D LV GLS, and 3D LV area strain are strong predictors of 60 months outcomes in patients with CD.

## Introduction

Chagas disease (CD) is an endemic and neglected infectious disease, first described in 1909 by Carlos Chagas^1^. Nowadays, more than a century after its description, it still remains associated with early mortality and substantial disability. Nevertheless, in recent decades, CD has increased worldwide and has become a health problem in non-endemic countries as a result of the migration of infected people^2,3^.

CD is characterized by two well established phases: an acute and a chronic phase. Approximately 20 to 40% of patients with CD will develop chronic Chagas cardiomyopathy (CCM), which is the most important and severe form of CD. Mortality due to CCM is closely related to the extent of cardiac function impairment and can be attributed to heart failure (HF), conduction disturbances, ventricular arrhythmias and sudden death and/or thromboembolism. CCM carries higher mortality risk than ischemic or idiopathic dilated cardiomyopathies^4–6^.

Despite the crucial importance of an adequate risk profile evaluation for therapeutic guidance, until now, prognostic markers have limited value^7,8^. In this context, echocardiography consists of a very useful tool not only for evaluation of LV function, but also for the analysis of other variables that can add incremental information and provide newer clinical perspectives for patients with CCM^9–14^.

More recently, techniques as three-dimensional (3D Echo) and speckle tracking echocardiography (STE) have become very useful and promising tools for the evaluation of cardiac mechanics, allowing a more precise and quantitative measurement of the global and regional myocardial longitudinal, radial and circumferential strain^15,16^. It is well established in literature that endocardial fibers, arranged longitudinally, are the first to undergo functional damage. Hence, longitudinal myocardial strain has been considered the best predictor of cardiac events in patients with HF, in comparison to left ventricular ejection fraction (LVEF) and Tissue Doppler data^15,16^.

Nevertheless, literature data are scarce concerning cardiac mechanics evaluation by 3D STE, particularly in patients with CD. The present study aims to clinically characterize new echocardiographic variables concerning cardiac mechanics according to LVEF and evaluate its role in the prediction of clinical outcomes in patients with CD.

## Methods

Eight hundred and eight patients with CD from the Cardiomyopathies and Heart Failure Clinics of a tertiary cardiological center were evaluated. Seventy-two consecutive patients with CD were recruited based on the following criteria: seropositivity for CD in two distinct laboratory tests (indirect hemagglutination assay, indirect immunofluorescence technique or ELISA—Enzyme-Linked Immunosorbent Assay) in sinus rhythm without other documented etiology. The exclusion criteria were age under 18 or beyond 70-years old, uncontrolled systemic arterial hypertension, diabetes mellitus, hypothyroidism, renal failure (serum creatinine > 1.5 mg/dL or glomerular filtration rate < 60 mL/min/1.73m^2^), hepatic failure, atrial fibrillation or frequent arrhythmias, coronary artery disease, patients with pacemakers, pregnancy and chronic obstructive pulmonary disease. This was a convenience sample because 3D STE is very sensitive technique and may be influenced by several clinical conditions.

All patients gave written informed consent, and the study was approved by the Hospital das Clínicas, Faculdade de Medicina, Universidade de Sao Paulo Ethical Committee. All patients underwent clinical, electrocardiographic and echocardiographic evaluation. All methods were performed in accordance with the relevant guidelines and regulations.

### Echocardiographic analysis

All studies were performed using the Vivid E9 (GE Healthcare Medical Systems, Milwaukee, WI, USA) with dedicated transthoracic transducers. Comprehensive conventional (2D Echo) and 3D Echo were performed according to joint recommendations from the American Society of Echocardiography^17^. Two and three-dimensional speckle tracking analysis were performed offline using a dedicated software (EchoPAC, BT12, GE Healthcare).

### Clinical follow-up

All patients were followed up 60 months. All patients were clinically evaluated by an assistant physician every 3 months or whenever necessary. Holter monitoring was assessed annually for each patient or whenever necessary. Patients were treated according the Brazilian Guidelines for CD^5^. Patients did not receive specific pharmacological treatment for *T.cruzi* infection. Composite endpoints were defined as hospitalization for heart failure (HF), complex ventricular arrhythmias (ventricular fibrillation and sustained ventricular tachycardia), heart transplant and death.

### Statistical analysis

Continuous numeric variables were expressed as mean ± standard deviation (SD). Normally distributed data were compared using a 2-sample Student’s t-test and 1-way analysis of variance. Non-normally distributed data were compared with Mann–Whitney U test and Kruskal–Wallis test. Categorical variables were expressed as frequency (percentage) and were compared using the chi-square test (x^2^) or the Fisher exact test, when appropriate. A two-tailed p value of 0.05 was considered significant.

Intraobserver and interobserver reproducibility of 2D STE parameters were evaluated in 10 randomly selected subjects and evaluated using concordance correlation coefficients (CCC) and Bland–Altman analysis. Intraobserver variability was assessed by having one observer re-measuring after 30 days. Another observer blinded to the first observer’s measurements evaluated the randomly selected exams for interobserver variability analysis. To determine the optimal cutoff value of prognostic STE parameters for predicting composite endpoints, mortality and hospitalization for heart failure, receiver-operating characteristic (ROC) curves were used.

Survival curves according to the LVEF were obtained in a Kaplan–Meier analysis and compared using the log-rank test. Uni and multivariate Cox regression models were used for estimations of the predictors of outcomes.

## Results

Eight-hundred and eight medical electronic reports of patients with CD followed in the single center where the study was performed were analyzed. Seventy-two patients were recruited according to the inclusion and exclusion criteria. The great majority of patients were excluded due to uncontrolled hypertension, diabetes mellitus, irregular cardiac rhythm, hypothyroidism and renal failure. Patients were enrolled in three groups: patients with LVEF < 0.40 (reduced LVEF, rLVEF) (N = 32); patients with LVEF ≥ 0.40 and ≤ 0.50 (mid-range or mildly reduced LVEF, mrLVEF) (N = 10); patients with LVEF > 0.50 (preserved LVEF, pLVEF) (N = 30).

### Clinical characteristics and conventional echocardiographic evaluation

Clinical, anthropometric and electrocardiographic data are described in Table 1. Conventional 2D Echo variables are comprised in Table 2. There were no difference between the groups regarding gender distribution, mean age and anthropometric variables.

**Table 1:** Clinical, anthropometric and electrocardiographic data.

	LVEF < 0.40 (N = 32)	LVEF ≥ 0.40 and < 0.50 (N = 10)	LVEF ≥ 0.50 (N = 30)	p
Gender	Male = 19 (59%)	Male = 7 (70%)	Male = 14 (47%)	0,71
Mean Age ± SD (years)	53.6 ± 9,1	48.0 ± 8.1	54.0 ± 9.4	0.17
Weight (Kg)	67.0 ± 9,9	71.8 ± 12.0	68.8 ± 10.9	0.45
Height (cm)	163.9 ± 7,7	164.1 ± 7.5	164.3 ± 7.2	0.98
BSA (g/m^2^)	1.73 ± 0.14	1.78 ± 0.18	1.75 ± 0.15	0.83
**Medical treatment**				
ACEi	N = 18 (56%)	N = 7 (70%)	N = 5 (17%)	**0.003**
ARBs	N = 11 (21%)	N = 3 (30%)	N = 5 (17%)	0.094
Beta blockers	N = 31(97%)	N = 10 (100%)	N = 7 (23%)	** < 0.001**
Furosemide	N = 22 (69%)	N = 3 (30%)	N = 0	** < 0.001**
Thiazide diuretics	N = 11 (21%)	N = 1 (10%)	N = 3 (10%)	0.005
Spironolactone	N = 19 (59%)	N = 7 (70%)	N = 0	** < 0.001**
Digoxin	N = 4 (13%)	N = 1 (10%)	N = 0	0.152
Nitrates	N = 5 (16%)	N = 0	N = 0	**0.002**
Hydralazine	N = 3 (9%)	N = 0	N = 0	0.109
Warfarin	N = 9 (28%)	N = 1 (10%)	N = 0	0.82
Amiodarone	N = 3 (9%)	N = 3 (30%)	N = 2 (7%)	0.62
**Functional class (NYHA)**				
I	N = 13 (41%)	N = 5 (50%)	N = 25 (83%)	** < 0.001**
II	N = 13 (41%)	N = 4 (40%)	N = 5 (17%)	
III	N = 5 (16%)	N = 1 (10%)	N = 0	
IV	N = 1 (2%)	N = 0	N = 0	
SBP (mmHg)	111.0 ± 12.3	116.2 ± 9.8	120.6 ± 12.7	**0.002**
DBP (mmHg)	71.2 ± 9.1	77.6 ± 7.2	78.1 ± 7.2	**0.004**
PR interval (ms)	0.18 ± 0.06	0.16 ± 0.03	0.17 ± 0.02	0.44
QRS interval (ms)	0.13 ± 0.03	0.12 ± 0.03	0.11 ± 0.03	0.06
**Morfology**				
LBBB	N = 4 (13%)	N = 0	N = 2 (7%)	0.33
RBBB	N = 12 (32%)	N = 3 (30%)	N = 2 (7%)	0.42
RBBB and left anterior fascicular block	N = 2 (6%)	N = 1 (10%)	N = 5 (17%)	0.32
**Holter**				
NSVT	N = 12 (38%)	N = 4 (40%)	N = 1 (3%)	0.05
Creatinine (mg/dl)	1.08 ± 0.27	0.98 ± 0.14	0.92 ± 0.2	**0.03**
Urea (mg/dl)	43.8 ± 15.6	35.7 ± 10.2	34.5 ± 11.4	**0.02**

Significant values are in bold.

*LVEF Left ventricular ejection fraction, *SD* Standard deviation, *BSA* Body surface area, *SAH* Systemic arterial hypertension, *ACEi* Angiotensin-converting-enzyme inhibitors, *ARBs* Angiotensin receptor blockers, *ASA* acetylsalicylic acid, *NYHA* New York Heart Association, *SBP* systolic blood pressure, *DBP* Diastolic blood pressure, *LBBB* Left bundle brunch block, *RBBB* Right bundle brunch block, *NSVT* Non-sustained ventricular tachycardia, *BNP* Brain natriuretic peptide.

**Table 2:** Conventional echocardiographic parameters.

	LVEF < 0.40 (N = 32)	LVEF ≥ 0.40 and < 0.50 (N = 10)	LVEF ≥ 0.50 (N = 30)	p
Aorta (mm)	31.4 ± 3.4	34.1 ± 4.1	32.3 ± 3.2	0.09
Left atrium (mm)	43.7 ± 7.4	40.7 ± 6.8	37.4 ± 5.0	**0.001**
Left atrium volume indexed (ml/m^2^)	49.5 ± 15.9	40.4 ± 11.2	30.6 ± 7.6	** < 0.001**
RA area (cm^2^)	18.9 ± 7.4	15.5 ± 2.5	15.3 ± 2.7	**0.02**
RA volume (cm^3^)	58.2 ± 39.0	42.2 ± 10.1	38.1 ± 12.0	**0.01**
RV basal diameter (mm)	37.3 ± 8.5	32.4 ± 5.5	32.5 ± 4.9	**0.01**
RV mid-level diameter (mm)	30.0 ± 7.4	25.8 ± 5.1	26.7 ± 4.4	**0.04**
LVDD (mm)	66.2 ± 7.2	59.7 ± 4.2	49.3 ± 4.2	** < 0.001**
LVSD (mm)	59.2 ± 8.1	49.2 ± 4.5	33.3 ± 5.2	** < 0.001**
RWT	0.24 ± 0.04	0.27 ± 0.03	0.34 ± 0.04	** < 0.001**
Myocardial ass index (g/m^2^)	134.9 ± 32.3	113.2 ± 18.3	85.4 ± 15.3	** < 0.001**
LVDVi (ml/m^2^)	117.4 ± 40.4	85.4 ± 22.1	55.0 ± 16.4	** < 0.001**
LVSVi (ml/m^2^)	82.7 ± 32.7	47.2 ± 14.1	21.2 ± 7.4	** < 0.001**
LVEF (Simpson method)	0.31 ± 0.06	0.45 ± 0.03	0.62 ± 0.05	** < 0.001**
TAPSE	14.7 ± 3.1	18.0 ± 4.1	18.4 ± 2.8	** < 0.001**
RV S´ wave	9.3 ± 2.2	10.2 ± 2.9	12.2 ± 2.0	** < 0.001**
FAC	0.42 ± 0.15	0.52 ± 0.07	0.51 ± 0.08	**0.004**
**Diastolic function**				
Normal	N = 0	N = 3 (30%)	N = 16 (53%)	** < 0.001**
Grade I	N = 8 (25%)	N = 6 (60%)	N = 14 (47%)	
Grade II	N = 10 (31%)	N = 1 (10%)	N = 0	
Grade III	N = 6 (19%)	N = 0	N = 0	
Grade IV	N = 7 (22%)	N = 0	N = 0	
Indeterminate	N = 1 (3%)	N = 0	N = 0	
Septal e´ velocity (cm/s)	4.9 ± 1.9	6.0 ± 2.4	7.9 ± 1.9	** < 0.001**
Lateral e´velocity (cm/s)	5.0 ± 2.3	8.0 ± 2.9	9.8 ± 3.2	** < 0.001**
Average E/e´	19.7 ± 12.4	10.2 ± 4.2	7.9 ± 2.0	** < 0.001**
**Mitral regurgitation**				
Absent	N = 5 (16%)	N = 2 (20%)	N = 23(77%)	** < 0.001**
Mild	N = 11 (34%)	N = 4 (40%)	N = 7 (23%)	
Moderate	N = 7 (22%)	N = 3 (30%)	N = 0	
Severe	N = 9 (28%)	N = 1 (10%)	N = 0	
**Aorticregurgitation**				
Absent	N = 26 (81%)	N = 7 (70%)	N = 26	0.28
Mild	N = 6 (19%)	N = 3 (30%)	N = 2	
Moderate	N = 0	N = 0	N = 0	
Severe	N = 0	N = 0	N = 0	
**Tricuspidregurgitation**				
Absent	N = 25 (78%)	N = 10 (100%)	N = 24 (86%)	0.06
Mild	N = 2 (6%)	N = 0	N = 4 (14%)	
Moderate	N = 1 (3%)	N = 0	N = 0	
Severe	N = 4 (13%)	N = 0	N = 0	
PASP (mmHg)	51.4 ± 14.4	NA	27.5 ± 7.5	**0.02**
TR peak velocity (m/s)	3.36 ± 0.40	NA	2.36 ± 0.28	**0.03**
Diffuse hypokinesis	N = 25 (78%)	N = 3 (30%)	N = 0	**0.02**
Inferior and lateral inferior walls akinesis	N = 3 (9%)	N = 3 (30%)	N = 2 (7%)	
Lateral inferior wall akinesis	N = 1 (3%)	N = 0	N = 0	
Apical akinesis	N = 2 (6%)	N = 2 (20%)	N = 0	
Apical aneurysm	N = 1 (3%)	N = 2 (20%)	N = 0	
Apical thrombus	N = 1 (3%)	N = 0	N = 0	0.36
**Pericardial effusion**				
Absent	N = 30 (94%)	N = 9 (90%)	N = 27 (95%)	0.67
Mild	N = 2 (6%)	N = 1 (10%)	N = 1 (5%)	

Significant values are in bold.

*LVEF Left ventricular ejection fraction, *SD* Standard deviation, *RA* right atrium, *RV* right ventricle, *LVDD* Left ventricle diastolic diameter, *LVSD* Left ventricle systolic diameter, *RWT* relative wall thickness, *LVDVi* Left ventricle diastolic volume indexed, *LVSDVi* Left ventricle systolic volume indexed, *TAPSE* Tricuspid annular plane systolic excursion, *FAC* Right ventricle fractional area change, *PASP* Pulmonary artery systolic pressure, *RT* Tricuspid regurgitation.

Bilateral cardiac chambers dimensions were larger in the rLVEF in comparison to the group with pLVEF. LV diastolic and systolic indexed volumes were greater in the group rLVEF than in the other groups. Thus, in these patients, LV indexed volumes were more accurate to distinguish the groups according to the LVEF than LV diameters and absolute volumes. Tricuspid annular plane systolic excursion (TAPSE) was the only RV systolic function parameter able to distinguish the groups. Septal e´ and lateral e´ velocities on Tissue Doppler were lower while E/e´ ratios were greater in the group rLVEF in G1 when compared to the other groups.

### Two-dimensional STE (2D STE)

2D STE data are shown in Table 3. 2D STE feasibility was very high in all the groups. LV GLS values were able to distinguish the groups according to the LVEF. RV GLS and RV free wall longitudinal strain values were lower in the group rLVEF in comparison to the other groups.

**Table 3:** Echocardiographic parameters of myocardial longitudinal, radial, circumferential strain and displacement evaluated by 2D STE.

	LVEF < 0.40 (rLVEF) (N = 32)	LVEF ≥ 0.40 and < 0.50 (mrLVEF) (N = 10)	LVEF ≥ 0.50 (pLVEF) (N = 30)	p
Heart rate (bpm)	62.3 ± 11.4	64.6 ± 12.0	62.2 ± 11.8	0.62
Frame Rate (qps)	59.9 ± 7.4	58.2 ± 5.1	60.4 ± 8.9	0.77
Feasibility	N = 572/576 (99.3%)	N = 178/180 (98.9%)	N = 504/504 (100%)	0.20
**LV global longitudinal strain**	− 9.2 ± 2.8	− 14.0 ± 3.4	− 19.1 ± 3.6	< 0.001 *, **, †
Apical 3-chambers GLS (%)	− 9.5 ± 3.5	− 13.9 ± 3.2	− 20.1 ± 4.1	** < 0.001** **, †
Apical 4-chambers GLS (%)	− 8.5 ± 3.3	− 13.4 ± 3.4	− 19.1 ± 4.2	** < 0.001** **, †
Apical 2-chambers GLS (%)	− 9.2 ± 3.3	− 13.3 ± 4.2	− 16.1 ± 7.8	< 0.001 *, **
**Basal (papillary muscles)**				
CS systolic peak (%)	− 7.3 ± 2.1	− 9.2 ± 1.9	− 12.3 ± 2.1	< 0.001 **, †
RS (%)	11.0 ± 6.5	13.9 ± 9.6	15.3 ± 9.1	0.12
Displacement (%)	3.2 ± 1.1	4.1 ± 0.8	5.0 ± 2.6	**0.002** *,**
Mid (mitral valve)				
CS Systolic peak (%)	− 7.5 ± 2.4	− 7.1 ± 4.3	− 11.5 ± 3.2	** < 0.001** **, †
RS (%)	13.4 ± 9.2	14.4 ± 6.0	28.7 ± 14.7	** < 0.001** **, †
Displacement (%)	3.3 ± 1.1	3.9 ± 0.8	5.1 ± 1.3	< 0.001 **, †
**Apical**				
CS Systolic peak (%)	− 9.1 ± 8.7	− 10.6 ± 2.3	− 15.0 ± 3.8	0.003 **, †
RS (%)	11.1 ± 7.6	15.5 ± 8.1	25.6 ± 20.8	0.001 **, †
Displacement (%)	2.5 ± 1.3	3.1 ± 0.9	4.2 ± 1.0	< 0.001 **, †
RV GLS	− 15.6 ± 5.2	− 21,5 ± 2.6	− 21 ± 3.4	< 0.001 *, **
RV FW longitudinal strain	− 18.8 ± 7.5	− 26.8 ± 3.9	− 24.1 ± 4.4	< 0.001 *, **

Significant values are in bold.

*LV Left ventricle, *RV* right ventricle, *GLS* Global longitudinal strain, *CS* Circumferential strain, *RS* Radial strain, *FW* free wall. *rLVEF × mrLVEF, p < 0.05, **rLVEF × pLVEF, p < 0.05, ^†^mrLVEF × pLVEF, p < 0.05. *rLVEF* reduced left ventricular ejection fraction, *mrLVEF* mid-range or mildly ejection fraction, *pLVEF* preserved left ventricular ejection fraction.

LV Peak systolic CS and LV displacement were lower in the group rLVEF in comparison to the group pLVEF. With exception of LV basal displacement, there were no differences regarding LV peak systolic CS and displacement between the groups rLVEF and mrLVEF. RV GLS and free wall strain were similar between the groups with mrLVEF and pLVEF.

### Three-dimensional echocardiographic results

**Figure 1:**
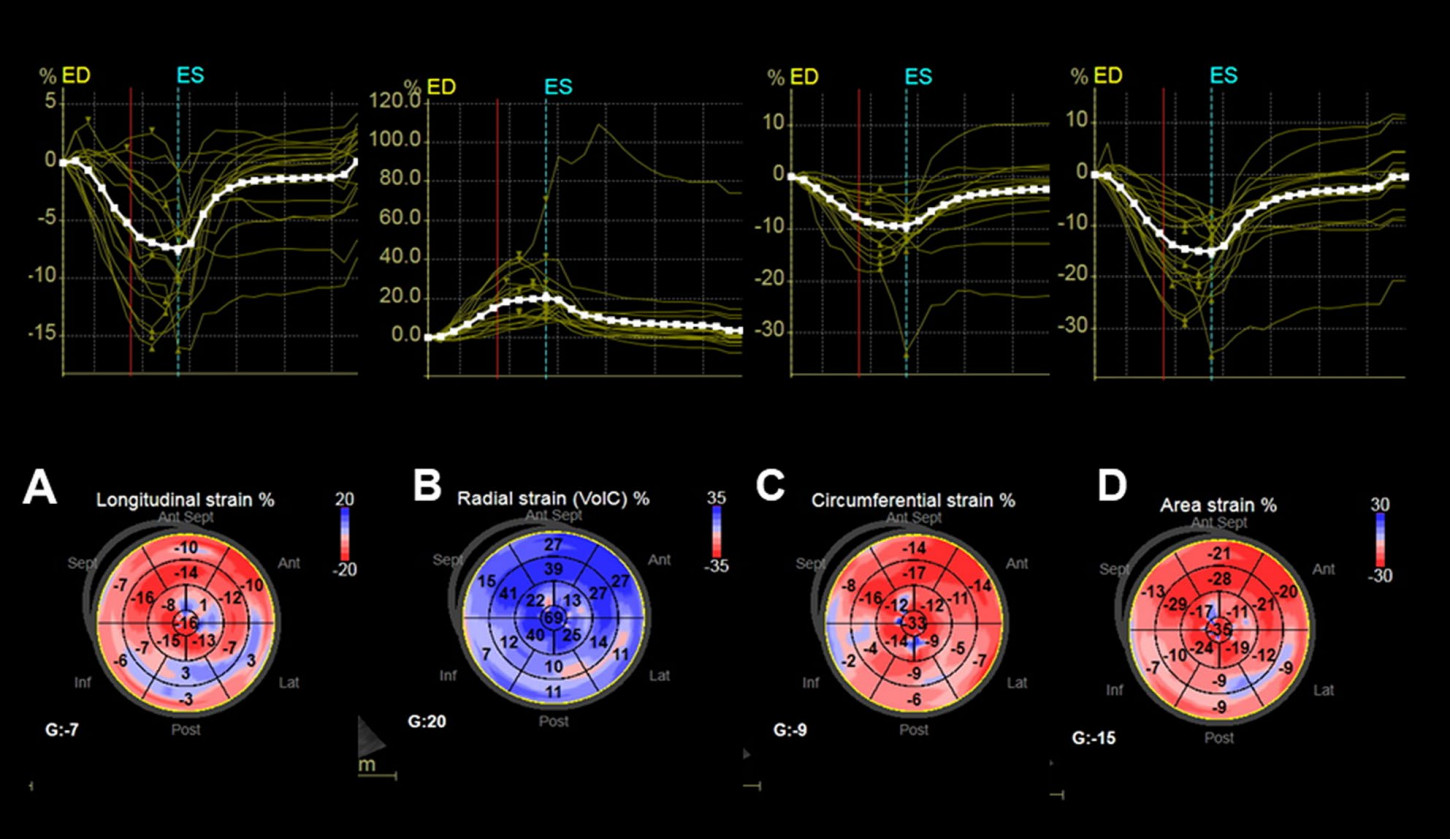
3D STE images from a patient with CD cardiomyopathy and severe LV dysfunction. (**A**) 3D LV global longitudinal strain = − 7%. (**B**) 3D LV global radial strain = 20%. (**C**) 3D LV global circumferential strain = − 9%. (**D**) 3D LV area strain = − 15%. 3D STE parameters are reduced diffusely. *3D STE* Three-dimensional speckle tracking echocardiography, *CD* Chagas disease, *LV* left ventricular.

3D Conventional Echo and STE parameters are shown in Table 4. There were no statistical differences between rLVEF and mrLVEF regarding left atrium (LA) indexed volume, LV end systolic indexed volume, sphericity index. 3D STE feasibility was very good in all the groups. GLS was different between the groups. There were no statistical differences between mrLVEF and pLVEF regarding 3D LV GLS, LV GCS, LV GCS or LV area strain. Figure 1 depicts 3D LV GLS, LV GRS, LV GCS and LV area strain of a patient with CD and severe LV dysfunction.

**Table 4:** Conventional and STE parameters by 3D Echo according to the LVEF.

	LVEF < 0.40 (rLVEF) (N = 32)	LVEF ≥ 0.40 and < 0.50 (mrLVEF) (N = 10)	LVEF ≥ 0.50 (pLVEF) (N = 30)	p
LA indexed volume (ml/m^2^)	37.6 ± 13.6	31.1 ± 10.1	21.3 ± 7.2	< 0.001 **, †
LVEDVi (ml)	114.3 ± 35.8	92.2 ± 16.5	58.0 ± 26.5	< 0.001 **, †
LVESVi (ml)	79.5 ± 28.3	50.6 ± 12.0	39.2 ± 10.5	** < 0.001** *, **, †
LVEF (%)	0.31 ± 0.06	0.46 ± 0.05	0.61 ± 0.03	** < 0.001** *, **, †
Sphericity index	0.48 ± 0.12	0.43 ± 0.08	0.36 ± 0.08	< 0.001 **, †
LV myocardial mass index (g/m^2^)	157.8 ± 50.9	139.3 ± 35.5	108.0 ± 27.5	< 0.001 **, †
Feasibility 3D STE	N = 527/576 (91.5%)	N = 161/180 (89.4%)	N = 421/476 (88%)	0.83
Global longitudinal strain (%)	− 9.0 ± 5.1	− 14.7 ± 3.7	− 16.8 ± 3.3	**0.001** *, **, †
Global circunferencial strain (%)	− 9.1 ± 3.9	− 12.5 ± 3.6	− 14.5 ± 6.9	**0.001** *, **
Global radial strain (%)	21.8 ± 11.0	37.6 ± 16.2	46.5 ± 11.5	** < 0.001** *, **
Área strain (%)	− 15.5 ± 6.8	− 24.6 ± 5.7	− 28.0 ± 5.2	** < 0.001** *, **

Significant values are in bold.

*LA Left atrium, *LVEF* Left ventricle ejection fraction, *LVEDVi* Indexed Left ventricle end diastolic volume, *LVESVi* Indexed Left ventricle end systolic volume. *rLVEF × mrLVEF, p < 0.05, **rLVEF × pLVEF, p < 0.05, †mrLVEF × pLVEF, p < 0.05. *rLVEF* reduced left ventricular ejection fraction, *mrLVEF* mid-range or mildly ejection fraction, *pLVEF* preserved left ventricular ejection fraction.

### Clinical follow up

**Figure 2:**
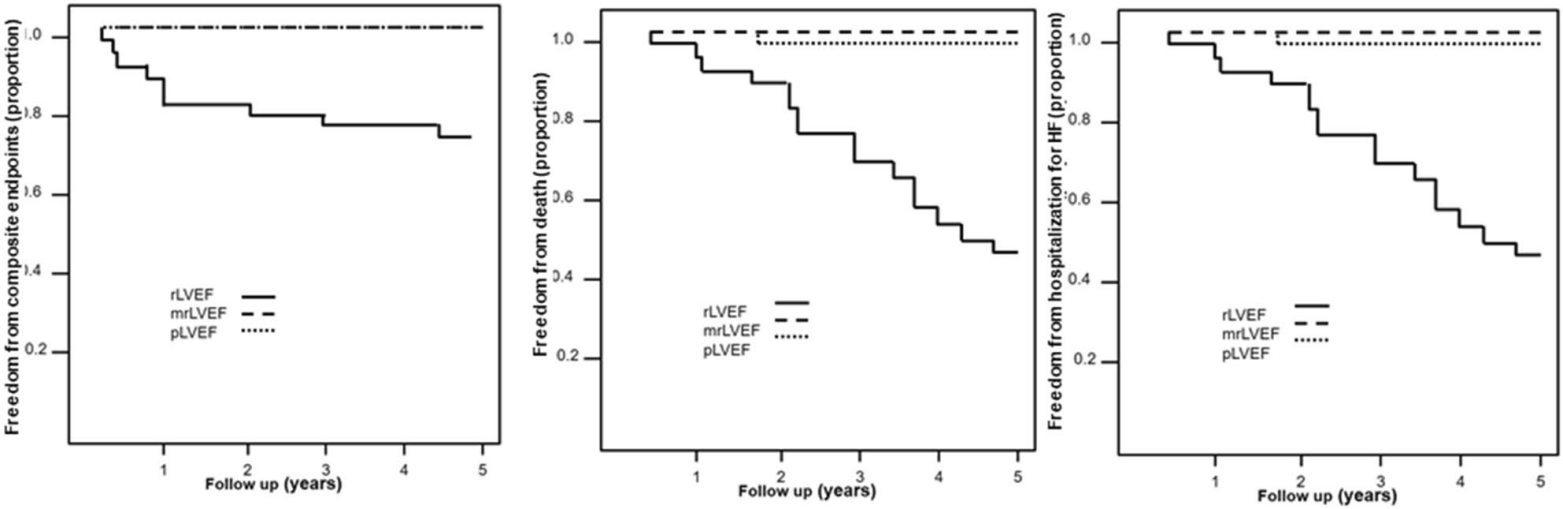
Composite endpoints (hospitalization for HF, complex ventricular arrhythmias, heart transplant and death), survival and hospitalization for heart failure Kaplan Meyer curves are shown for the three groups. *HF* heart failure, *rLVEF* reduced left ventricular ejection fraction, *mrLVEF* mid-range or mildly ejection fraction, *pLVEF* preserved left ventricular ejection fraction.

All patients were followed up for 60 months. Composite endpoints (hospitalization for HF, complex ventricular arrhythmias, heart transplant and death), survival and hospitalization for heart failure Kaplan Meyer curves are shown in Fig. 2. One patient in pLVEF group died of non-cardiovascular cause. There were no clinical events in mrLVEF. There were nine hospitalizations for decompensated HF, two episodes of complex ventricular arrhythmias, two heart transplants and seventeen deaths in rLVEF.

#### Composite endpoints

In the overall patients, bilateral cardiac remodeling was associated with composite endpoints. Larger atrial and ventricular diameters and volumes (absolute or indexed) as well as reduced biventricular function evaluated by 2D and 3D Echo, reduced velocities at tissue Doppler and increased E/e´ ratio were associated with composite endpoints. 2D LV GLS, 2D LV GCS, RV GLS, 3D LV GLS and 3D LV area strain were the strongest predictors of composite endpoints (Table 5).

**Table 5:** Predictors of Composite Endpoints, overall mortality and hospitalization for heart failure in patients with Chagas Disease.

Variable	Hospitalization for Heart Failure			Death			Composite Endpoints		
	p	HR	95% CI	p	HR	95% CI	p	HR	95% CI
LA Diameter	0.002	1.188	1.063–1.328	0.015	1.093	1.018–1.174	< 0.001	1,121	1,055–1,192
LA volume	< 0.001	1.060	1.030–1.092	< 0.001	1.026	1.011–1.041	< 0.001	1,027	1,015–1,040
LA indexed volume (ml/m^2^)	< 0.001	1.061	1.03–1.092	< 0.001	1.052	1.025–1.087	< 0.001	1,05	1,028–1,072
RA area (cm^2^)	< 0.001	1.183	1.086–1.289	< 0.001	1.144	1.070–1.224	< 0.001	1,206	1,12–1,29
RA volume (ml)	< 0.001	1.030	1.015–1.045	< 0.001	1.026	1.013–1.039	< 0.001	1,036	1,022–1,049
TAPSE (mm)	0.12	0.853	0.699–1.041	0.02	0.850	0.740–0.976	0.009	0,854	0,76–0,96
RV S´ wave (cm/s)	0.006	0.682	0.521–0.894	0.05	0.823	0.678–1.000	0.003	0,784	0,669–0,92
FAC	0.002	0.001	0.000–0.064	0.06	0.040	0.001–1.070	0.001	0,008	0,001–0,128
LVDD (mm)	< 0.001	1.147	1.063–1.238	< 0.001	1.123	1.063–1.186	< 0.001	1,167	1,107–1,229
LVSD (mm)	0.001	1.118	1.044–1.197	< 0.001	1.104	1.055–1.156	< 0.001	1,123	1,077–1,171
RWT	0.001	0.000	0.000–0.000	< 0.001	0.000	0.000–0.003	< 0.001	0.000	0,00–0,00
LVEDV (ml)	0.001	1.011	1.005–1.017	< 0.001	1.011	1.005–1.016	< 0.001	1,013	1,009–1,017
LVESV (ml)	< 0.001	1.013	1.006–1.021	< 0.001	1.013	1.007–1.019	< 0.001	1,016	1,011–1,021
Indexed Myocardial Mass (g/m^2^)	0.025	1.021	1.003–1.039	< 0.001	1.026	1.013–1.040	< 0.001	1,027	1,016–1,038
LVEF (Simpson)	0.004	0.000	0.000–0.031	< 0.001	0.000	0.000–0.014	< 0.001	0.000	0,000–0,003
LVDV indexed (ml)	0.002	1.017	1.007–1.029	< 0.001	1.020	1.010–1.030	< 0.001	1,021	1,014–1,029
LVSV indexed (ml)	< 0.001	1.022	1.010–1.034	< 0.001	1.023	1.012–1.034	< 0.001	1,026	1,017–1,035
E wave (cm/s)	0.001	1.051	1.02–1.084	0.002	1.038	1.013	< 0.001	1,038	1,018–1,059
A wave (cm/s)	0.032	0.942	0.893.0.995	0.06	0.972	0.943–1.001	0.021	0,97	0,945–0,995
Septal e´ (cm/s)	0.021	0.65	0.451–0.937	0.002	0.692	0.546–0.875	0.001	0,713	0,587–0,867
Lateral e´ (cm/s)	0.018	0.732	0.564–0.948	< 0.001	0.694	0.574–0.839	< 0.001	0,714	0,609–0,836
Mean E/e´ratio	< 0.001	1.076	1.035–1.119	< 0.001	1.055	1.025–1.085	< 0.001	1,059	1,033–1,086
Global RV longitudinal strain (%)	0.002	1.220	1.075–1.384	0.001	1.152	1.056–1.257	< 0.001	1,158	1,076–1,245
RV free wall Longitudinal strain (%)	0.02	1.048	1.0 – 1.099	0.04	1.04	1.002–1.079	0.04	1.037	1.005–1.070
2D GLS (%)	< 0.001	1,493	1.199–1,858	< 0.001	1.242	1.116–1.381	< 0.001	1,317	1,184–1,465
2D GCS (%)	0.008	1,427	1.097–1.857	< 0.001	1.447	1.197–1.750	< 0.001	1,329	1,147–1,539
2D GRS (%)	0.98	1,001	0,932–1,075	0.38	0.975	0.92–1.032	0,92	0,998	0,957–1,041
3D LA indexed volume (ml)	< 0.001	1,075	1.033–1.118	< 0.001	1.058	1.029–1.088	< 0.001	1,054	1,029–1,079
3D LVEF (%)	0.004	0,000	0.000–0.032	< 0.001	0.000	0.000–0.008	< 0.001	0.000	0,000–0,002
3D GLS (%)	0.012	1,146	1,03–1,275	< 0.001	1.197	1.090–1.314	< 0.001	1,186	1,102–1,276
3D GCS (%)	0.09	1,066	0,99–1,147	0.005	1.076	1.022–1.132	0.01	1,062	1,015–1,112
Area Strain (%)	0.009	1,107	1,026–1,194	< 0.001	1.155	1.076–1.239	< 0.001	1,123	1,067–1,083
3D GRS	0.009	0,933	0,885–0,992	< 0.001	0.914	0.871–0.959	< 0.001	0,932	0,901–0,964

*HR* hazard ratio, *CI* confidence interval. *Values of p < 0.05 indicate statistical significance.

*FAC* fractional area change, *LA* left atrium, *LV* left ventricle, *LVDD* left ventricular diastolic diameter, *LVDV* left ventricular diastolic volume, *LVEDV* left ventricular end-diastolic volume, *LVEDV* left ventricular end-systolic volume, *LVEF* left ventricular ejection fraction, *LVSD* left ventricular systolic diameter, *LVDV* left ventricular diastolic diameter, *RA* right atrium, *RV* right ventricle, *RWT* relative wall thickness, *TAPSE* tricuspid annular plane systolic excursion.

#### Death from all causes and hospitalization for heart failure

As for composite endpoints, bilateral cardiac remodeling was associated with mortality. Larger atrial and ventricular diameters and volumes (absolute or indexed) as well as reduced LV and RV function (TAPSE) evaluated by 2D and 3D Echo, reduced velocities at tissue Doppler and increased E/e´ ratio were associated with death from all causes and hospitalization for heart failure. Interestingly, RV S´ wave, FAC were not associated with overall mortality.

2D LV GLS, 2D LV GCS, RV GLS, 3D LV GLS and 3D LV area strain were the strongest predictors of death from all causes and hospitalization for HF (Table 5).

#### ROC curves for prediction of cardiovascular outcomes

**Figure 3:**
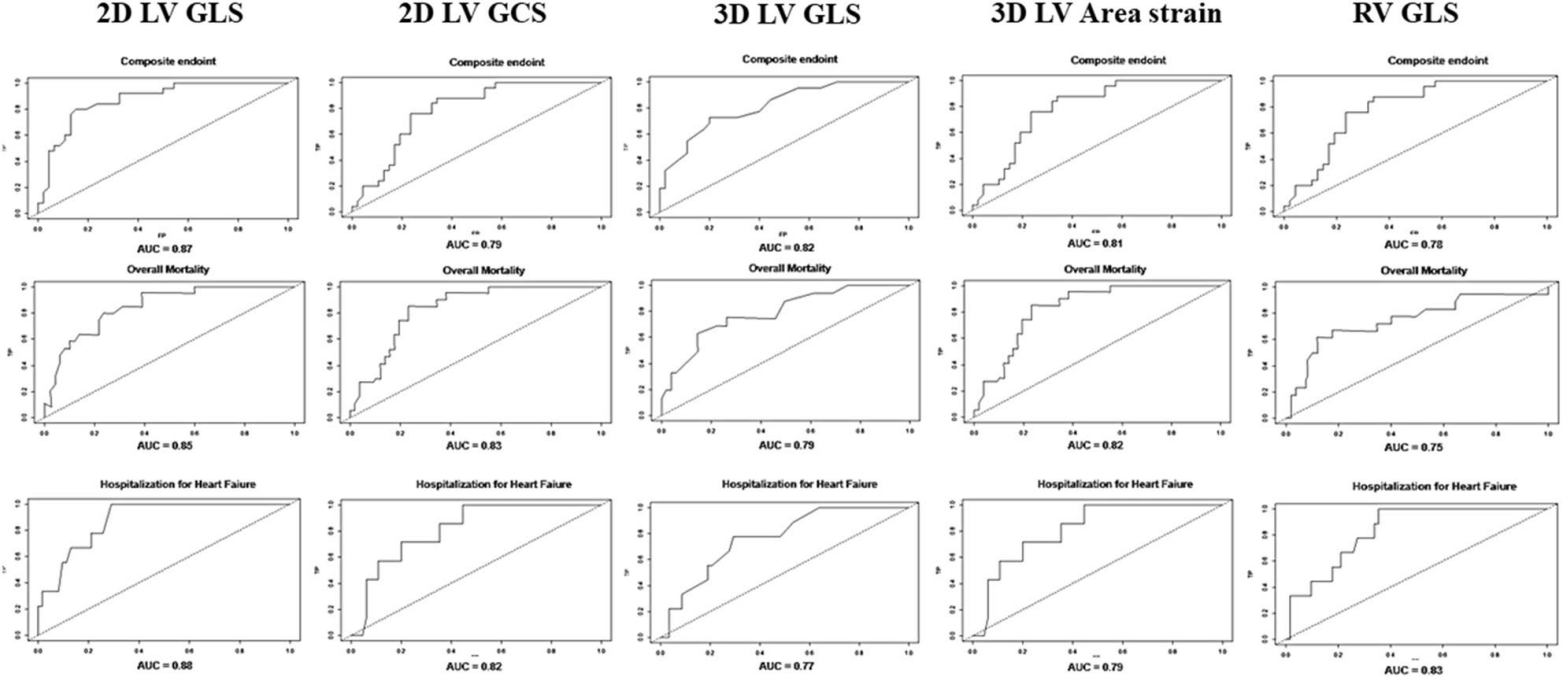
Receiver-operator characteristic (ROC) curves of the strong predictors of cardiovascular events during the 5 years of follow up. *2D LV GLS* Two-dimensional left ventricular global longitudinal strain, *2D LV GCS* Two-dimensional left ventricular global circumferential strain, *3D LV GLS* Three-dimensional left ventricular global longitudinal strain, *3D LV Area strain* Three-dimensionalleft ventricular area strain, *AUC* area under curve, *RV GLS* Right ventricular global longitudinal strain.

Receiver-operator characteristic (ROC) curves of the strong predictors of cardiovascular events were built for the 5 years of follow up (Fig. 3). The cut offs of the STE parameters that yield the prediction of outcomes in 5 years of follow up are depicted in Table 6.

**Table 6:** STE parameters´ cut off values for prediction of events in 5 years of follow up.

	Death	Hospitalization	Composite endpoints
2D GLS	− 14,4	− 11,3	− 11,3
2D GCS	− 8,6	− 9,8	− 10,1
3D GLS	− 10,0	− 12,0	− 13,0
AreaStrain	− 16,0	− 15,0	− 16,0
RV GLS	− 14,9	− 19,4	− 17,2

*2D GLS* Two-dimensional global longitudinal strain, *2D GCS* Two-dimensional global circumferential strain, *3D GLS* Three-dimensional global longitudinal strain, *RV GLS* Right ventricular global longitudinal strain.

There is known collinearity between the LVEF obtained from the conventional 2D Echo evaluation and the cardiac mechanic parameters derived from the STE analysis and 3D Echo data. Also, it was observed non-linear relation of LVEF according to the patients´ age. So, we presented the estimated relation of STE analysis and 3D Echo data individually adjusted for age and cubic natural splines of LVEF (Table 7).

**Table 7:** Estimated relation of STE analysis and 3D Echo data individually adjusted for age and cubic natural splines of LVEF.

	Death	Hospitalization	Composite endpoints
2D GLS	1.14 [0.93–1.4], p = 0.207	**1.74 [1.14–2.66], p = 0.01**	1.16 [0.95–1.42], p = 0.135
2D GCS	1.16 [0.92–1.48], p = 0.211	1.04 [0.69–1.57], p = 0.835	0.9 [0.72–1.11], p = 0.331
3D GLS	1.08 [0.95–1.24], p = 0.237	0.94 [0.75–1.16], p = 0.549	1.05 [0.93–1.18], p = 0.416
LA	1.03 [0.94–1.13], p = 0.497	**1.53 [1.12–2.1], p = 0.008**	1.06 [0.97–1.15], p = 0.213
3D LA indexed volume	1.03 [0.99–1.07], p = 0.147	**1.1 [1–1.21], p = 0.044**	1.02 [0.99–1.05], p = 0.26
LVDD	**1.1 [1.01–1.2], p = 0.024**	1.09 [0.96–1.24], p = 0.185	**1.09 [1.02–1.18], p = 0.018**
LVSD	**1.1 [1–1.21], p = 0.047**	**1.14 [1–1.31], p = 0.047**	**1.11 [1.03–1.21], p = 0.01**
RV GLS	1.1 [0.99–1.23], p = 0.09	1.18 [0.95–1.47], p = 0.134	1.06 [0.96–1.16], p = 0.246
Area Strain	1.09 [0.99–1.2], p = 0.066	0.98 [0.84–1.13], p = 0.755	1.02 [0.93–1.12], p = 0.717

Significant values are in bold. *2D GLS* Two-dimensional global longitudinal strain, *2D GCS* Two-dimensional global circumferential strain, *3D GLS* Three-dimensional global longitudinal strain, *LA* Left atrium, *3D LA indexed volume* Three-dimensional left atrium indexed volume, *LVDD* Left ventricular diastolic diameter, *LVSD* Left ventricular systolic diameter, *RV GLS* Right ventricular global longitudinal strain.

For prediction of death, LVDD and LVSD evidenced incremental value over LVEF; for prediction of hospitalization, 2D GLS, LA diameter, 3D LA indexed volume and LV DD evidenced incremental value over LVEF and for composite endpoints, LVDD and LVSD evidenced incremental value over LVEF.

#### Reproducibility

The intraobserver and interobserver reproducibility of most of the STE parameters was excellent, as reflected by high CCC (Supplemental File; Table 1), with exception of 2D LV GCS and 2D LV GRS. Bland–Altman analysis demonstrated good intraobserver and interobserver agreement (Supplemental File; Table 2).

## Discussion

To the best of our knowledge, this is the first study to evaluate 3D conventional and strain analysis in patients with CD from normal LVEF up to reduced LVEF. So far, only 2D STE parameters have been described in different stages of CD and most studies evaluated patients with normal LVEF^18–21^. Very few studies have reported 3D Echo conventional parameters in patients with CD but none have included 3D STE analysis^22–24^. The main findings of our study were: (1) 3D conventional and 2D STE analysis in mrLVEF were anatomically and functionally similar to rLVEF; (2) RV GLS, 2D LV GLS, 2D LV GCS, 3D LV GLS, and 3D LV area strain were strong predictors of outcomes in patients with CD, with superior value to other conventional parameters provided by cardiac dimensions, Tissue Doppler indexes or TAPSE and fractional area change for prediction of overall mortality or composite endpoints.

Our results are in disagreement with previous studies suggesting that in patients with the indeterminate form of CD with pLVEF, global and segmental longitudinal systolic strain is reduced compared with healthy subjects, thus indicating that it could be a sensitive technique to detect early myocardial damage^19,21^. In other published investigation, only regional LV longitudinal strain was reduced in indeterminate form of CD with pLVEF that is in concordance with our results. Also, in a recent study, with patients in different CCM stages, 2D LV GLS was the more accurate measurement regarding stage A differentiation from the stages B, C, and D^25^. However, reports of 2D LV GLS in CD are conflicting and still remain a controversial issue. To explain the abnormal strain, myocarditis and inflammatory infiltrate was found in 15% and 37% respectively of indeterminate phase^26^. In another publication, in 60.6% of patients with indeterminate phase, it was found abnormalities like fiber degeneration, volume changes, interstitial edema, inflammatory infiltrates and fibrosis^27^. Besides, it was reported even in patients with Chagas disease with preserved or minimally impaired ventricular function cardiac fibrosis in 3% and 11% respectively using late gadolinium enhancement on cardiac magnetic resonance (CMR)^28^. Moreover, some studies support the use of 2D LV GLS in surveillance providing a window of opportunity for early intervention and preventing heart failure patients.

Patients with indeterminate form of CD are defined as patients seropositive for CD but with normal electrocardiogram and normal LVEF. These patients have a very benign clinical course that may be compared with matched cohorts of patients without cardiac disease. Some studies suggest that these patients may have subclinical of subtle cardiac disease that may be identified with STE techniques. Nevertheless, in our study we did not confirm these findings making it necessary more studies to determine the practical use of STE in these patients. These controversial data may be due to the limited number of studies and patients for this purpose allied with the ongoing developing state of echocardiographic techniques.

In our study, 3D conventional Echo and 2D STE parameters evidenced similar results comparing rLVEF and mrLVEF. Hitherto, the pathophysiology of rLVEF and mrLVEF heart failure patients is incompletely understood and, consequently, the reasons for this finding are unclear. In other etiologies, mrLVEF patients may include a heterogeneous population with patients that partially recovered the cardiac function under guidelines-directed medical treatment, or patients that have not yet evolved to rLVEF, or patients that never will follow to rLVEF. Previously, we reported improvement in CCM under guidelines-directed medical treatment^28^. It was reported in reduced and mid-range LVEF HF patients similar natriuretic peptide elevated levels, and neuroendocrine profile. However, cardiac troponin values in mid-range LVEF HF patients are intermediate to those with reduced and preserved LVEF HF patients. On other hand, studies on endomyocardial biopsies from CD patients showed that the clinical evolution of the disease was correlated with a continuous progression of fiber destruction, fibrosis, myocardial inflammation, and a reduction in performance^2^. These findings may indicate that, possibly, patients with CD mrLVEF could be sharing with rLVEF some pathophysiological mechanisms related to 3D conventional Echo and 2D STE parameters but for all characteristics. In fact, a recent meta-analysis concluded that significant differences in hospitalization and mortality were detected between mildly or mid-range heart failure and the other subtypes of heart failure including diverse etiologies but not included CD^29^. Nevertheless, CD is a very peculiar etiology that is not included in the studies. Herein, there is an extreme paucity of data concerning patients with mildly or mid-range Chagas heart disease and no study so far evaluated 3D cardiac mechanics in patients with mrLVEF and CD.

Our findings that RV GLS, 2D LV GLS, 2D LV GCS, 3D LV GLS, and 3D LV area strain were strong predictors of outcomes in patients with CD are in concordance with an increasing number of studies that have suggested that 2D LV GLS is superior to LVEF as a measure of LV function and as a predictor of mortality and cardiac events in other etiologies, mainly ischemic cardiac disease^30^. After adjustment for LVEF, there were no differences in STE values between Chagas Disease cardiomyopathy and idiopathic cardiomyopathy in reduced LVEF. 2D LV GLS was a strong predictor of adverse events, incremental to LVEF and E/e' ratio in dilated cardiomyopathy that included also chagasic etiology. No absolute values for 2D LV GLS indicating high risk are established, but a value of 2D LV GLS of − 12% has been suggested representing severe systolic dysfunction and adverse prognosis^30^. Our findings are in concordance with this value and a value of 2D LV GLS lower than − 11.4% was associated with composite endpoints. 2D LV GCS is not well established as a predictor of outcomes because 2D LV GLS is the most robust parameter for this purpose but in our study, a value of 2D LV GCS lower than − 10.1% was also associated with composite endpoints.

The value of 2D LV GLS as a predictor of outcomes relies on the hypothesis that this parameter reflects changes in the myocardial interstitium yielding information regarding the extent of myocardial fibrosis as suggested by findings in patients with mitral regurgitation and hypertrophic cardiomyopathy. In another study, both GLS and ejection fraction were significant predictors of myocardial fibrosis. It was observed a high correlation with both ejection fraction assessed by echocardiography (*r* = 0.70, *p* < 0.001) and GLS (*r* = 0.64, *p* < 0.001) regarding the percentage of fibrosis. Nevertheless, after multiple linear regression analysis, the 2D LV GLS were no longer a predictor of myocardial fibrosis. So, the authors concluded in this study that 2D LV GLS has no incremental value to left ventricular ejection fraction assessed by conventional echocardiography in the prediction of myocardial fibrosis in patients with Chagas disease^31^.

In respect to the RV GLS, several studies showed that this parameter provides strong additional prognostic value to predict overall and cardiovascular mortality in rLVEF patients with other etiologies, essentially ischemic cardiac disease. The predictive value was even higher than parameters evaluated by CMR as RV ejection fraction and RV strain^32^. In concordance in our study, RV GLS was also a strong predictor of outcomes. Values of RV GLS under − 17.2% and − 14.9% were associated with composite endpoints and mortality, respectively. Similarly, an RV GLS under − 19% was related to all-cause or cardiovascular mortality in rLVEF patients with ischemic, hypertensive or idiopathic cardiomyopathies^32^. As shown in previous studies in other etiologies and potentially also for CCM, RV GLS and RV free wall LS have performed better than conventional parameters for the prediction of outcomes. This may be probably because the use of the longitudinal strain for RV as for LV evaluation allows the analysis of the deformation of the endocardial fibers, which might be more sensitive to reduced coronary perfusion and increased wall stress and are usually affected earlier in myocardial diseases.

Concerning 3D analysis, our findings that 3D LV GLS and 3D LV area strain are strong predictors of outcomes in CCM suggest a very innovative clinical application for this technique. Until now, some studies evaluated the prognostic value of 3D Strain in a variety of clinical scenarios including valvular heart disease, ischemic heart disease and chronic renal failure, showing that the reduction of 3D values was associated with poor outcomes^33^. Particularly, 3D LV area strain is a very promising index that quantifies endocardial area change, integrating longitudinal and circumferential deformation, allowing a more detailed evaluation of the different types of myocardial fibers and enabling a better understanding of the pathophysiology of cardiomyopathies^32,33^. In a recent meta-analysis that aimed to determine normal ranges of 3D Strain, the authors reported that the mean value of 3D LV GLS was 19.1%, ranging from 15.8 to 23.4% among the studies^34^. In our study, 3D LV GLS values and 3D LV area strain under − 13% and − 10%, were associated with composite endpoints and mortality, respectively.

### Perspectives

The use of new technologies for better understanding CD presents a great potential for clinical application. The evaluation of cardiac mechanics can be performed non-invasively, with practically no risk. Our study suggests that 2D and 3D strain analysis should be added to the comprehensive evaluation of patients with CD and rLVEF for prognostication. Our findings reinforce and extend previous studies´ results showing that RV GLS, 2D, and 3D STE may provide additional information for better risk assessment of these patients with high feasibility and good intra and interobserver variability.

Although the application of strain imaging has yet not been included in clinical practice guidelines, it is likely to become a useful application when evaluating patients with CD.

### Clinical implications

In our study, it was observed high feasibility of 2D and 3D strain analysis in all the groups. Despite the significant LV dilatation, especially in the group with rLVEF, which could limit the analysis of all LV segments, the feasibility was close to 100%. These findings demonstrate the great applicability of this technique, even in patients with extensive ventricular remodeling and/or presence of abnormalities in the apical region, very frequent in patients with CD^14^.

The finding of better correlation of RV GLS, 2D LV GLS, 2D LV GCS, 3D LV GLS, and 3D LV area strain with outcomes than conventional 2D and 3D Echo conventional indexes in patients with CD provides important and potentially incremental information that may contribute to the establishment of cutoff values ​​for different degrees of LV dysfunction and prognosis in addition to the already established values ​​of LVEF. Therefore, we hypothesize that, eventually, the analysis of biventricular longitudinal strain could add incremental data regarding mortality and composite endpoints, predicting outcomes in patients with CD, as previously described, shedding light on complexes mechanisms of cardiomyocytes contraction imbalance and pathophysiology of patients with Chagas cardiomyopathy as a useful tool for prognostication.

Thereby, cardiac mechanics provide more refined and accurate information related to cardiac dysfunction and derangement than conventional parameters expressed by morphological variables as cardiac diameters and volumes and LVEF and might be related to myocardial fibrosis as evaluated by CMR but with expressive lower cost, mainly considering the economic issues in the developing countries, where CD is more prevalent.

## Limitations

Due to the high prevalence of unknown or poorly controlled hypertension, in addition to diabetes mellitus and hypothyroidism, the vast majority of patients could not be included in this study, limiting the final number of patients. However, this extreme caution is mandatory when evaluating new techniques, without definite cutoff values and clinical applications. The small number of patients is a limitation as well as the fact that this was a single-center study. Also, there was an imbalance between the three groups analyzed because the study included a convenience sample. Another limitations concern the limited availability of specific equipment besides more substantial time to acquire the datasets and perform all the measurements. Currently, conventional 3D Echo and 3D STE are available in a very limited number of research centers of echocardiography. Besides, both techniques require a learning curve and expertise until an adequate examination is possible. Nowadays, there is still inter-vendor variability in 3D STE measurements and spatial and temporal resolution are not yet adequate to perform reliable segmental analysis. Finally, there still considerable interobserver variability of 3D strain measurements but that was not a limitation in this study.

## Conclusions

Patients with CD and mrLVEF presented anatomic and functional characteristics more similar to the CD and rLVEF despite the benign clinical evolution similar to patients with pLVEF. RV GLS, 2D LV GLS, 2D LV GCS, 3D LV GLS, and 3D LV area strain are strong predictors of outcomes in patients with CD. These findings suggest that 2D and 3D strain yields a potential incremental value for clinical decision guidance in patients with Chagas heart Disease.

## Supplementary Information

Supplementary Information.

## Abbreviations

2D: Two-dimensional
3D: Three-dimensional
CD: Chagas disease
GCS: Global circumferential strain
GLS: Global longitudinal strain
GRS: Global radial strain
HF: Heart failure
LV: Left ventricular
LVEF: Left ventricular ejection fraction
mrLVEF: Mildly reduced left ventricular ejection fraction
pLVEF: Preserved left ventricular ejection fraction
rLVEF: Reduced left ventricular ejection fraction
RV: Right ventricular
STE: Speckle tracking echocardiography
TAPSE: Tricuspid annular plane systolic excursion
Echo: Echocardiography
