# Supplementary material for: Three and two-dimensional cardiac mechanics by speckle tracking are predictors of outcomes in chagas heart disease
Source: Sci Rep. 2022 Jul 18;12:12237. doi: 10.1038/s41598-022-16379-w (PMC9293971; doi:10.1038/s41598-022-16379-w)
Supplement: Supplementary file 1 — Supplementary Information. [file 41598_2022_16379_MOESM1_ESM.docx]

Table 1. CCC analysis concerning intraobserver and interobserver reproducibility of the 2D and 3D STE parameters.

|  | **INTRAOBSERVER** | | **INTEROBSERVER** | |
| --- | --- | --- | --- | --- |
|  | **C.C.C.** | **C.B.** | **C.C.C.** | **C.B.** |
| 2D GLS | 0.98 | 0.99 | 0.90 | 0.96 |
| 2D GCS | 0.62 | 0.95 | 0.57 | 0.70 |
| 2D GRS | 0.37 | 0.78 | 0.24 | 0.54 |
| 2D Displacement | 0.93 | 0.96 | 0.54 | 0.78 |
| 3D GLS | 0.92 | 0.95 | 0.88 | 0.96 |
| 3D Area Strain | 0.95 | 0.98 | 0.90 | 0.98 |
| 3D GRS | 0.87 | 0.92 | 0.83 | 0.93 |
| 3D GCS | 0.92 | 0.97 | 0.81 | 0.98 |

2D GLS, Two-dimensional global longitudinal strain; 2D GCS, Two-dimensional global circumferential strain; 2D GRS, Two-dimensional global radial strain; 3D GLS, Three-dimensional global longitudinal strain; 3D GCS, Three-dimensional global radial strain; 3D GRS, Three-dimensional global circumferential strain. CCC, Lin's concordance correlation coefficient (CCC); CB, accuracy.

Table 2. Bland-Altman analysis concerning intraobserver and interobserver reproducibility of the 2D and 3D STE parameters.

|  | **INTEROBSERVER** | **INTRAOBSERVER** |
| --- | --- | --- |
| **2D GLS** | 0.96 ± 0.04 | 0.90 ± 0.08 |
| **3D GLS** | 0.92 ± 0.07 | 0.93 ± 0.10 |
| **3D GCS** | 0.88 ± 0.07 | 0.82 ± 0.15 |
| **3D AS** | 0.93 ± 0.05 | 0.90 ± 0.05 |
| **3D RS** | 0.85 ± 0.05 | 0.84 ± 0.11 |

2D GLS, Two-dimensional global longitudinal strain; 2D GCS, Two-dimensional global circumferential strain; 2D GRS, Two-dimensional global radial strain; 3D GLS, Three-dimensional global longitudinal strain; 3D GCS, Three-dimensional global radial strain; 3D GRS, Three-dimensional global circumferential strain.
